# Underwater multi-sensor information fusion for salient object detection

**DOI:** 10.1371/journal.pone.0354804

**Published:** 2026-08-06

**Authors:** Yan Mou, Zhaolong Gao, Jinjiang Li

**Affiliations:** School of Computer Science and Technology, Shandong Technology and Business University, Yantai, Shandong, China; Northwest Normal University, CHINA

## Abstract

Underwater salient object detection is a critical task in computer vision, relying heavily on data from underwater sensors, with wide-ranging applications in object tracking, content-aware editing, and object recognition. To tackle the challenges inherent in underwater multimodal information fusion, this paper introduces a novel underwater salient object detection framework based on an information cross-fusion network. The proposed approach integrates a cross-attention feature injection module and an information embedding module to facilitate efficient multimodal feature aggregation and refinement across both channel and spatial dimensions. By modeling the complementarity between RGB and depth data at global and local scales, these modules enhance the representation of salient regions while effectively suppressing background noise. Furthermore, the architecture employs multi-level and multimodal information fusion, which mitigates the effects of depth-related noise and reduces uncertainty in predictions. Extensive experiments conducted on multiple underwater datasets demonstrate that the proposed method achieves superior performance compared to state-of-the-art approaches, highlighting its efficacy in multimodal feature integration and salient object detection.

## Introduction

Underwater salient object detection aims to identify the most conspicuous objects or regions in underwater images [1–3]. Underwater salient object detection faces unique challenges, including light attenuation, color distortion, water scattering, and the blurring of object shapes and textures [4]. Meanwhile, the reliance on data from underwater sensors, such as RGB cameras and depth sensors, introduces complexities related to sensor noise and modality misalignment. Effectively addressing these challenges requires robust fusion techniques to integrate information from multi-sensor.

With the advancement of deep learning techniques, RGB-based underwater object detection has achieved improvements in both accuracy and speed [5–7]. With the advancement of data-driven approaches, the construction of the USOD10K dataset —the first large-scale underwater RGB-D benchmark—has provided a solid foundation for training robust models across diverse and complex scenarios. Building upon this, researchers have explored more sophisticated fusion architectures. For instance, Wang et al. proposed [8], which utilizes a hierarchical cross-modality attention network to effectively suppress underwater noise and generate more accurate saliency maps by refining multi-modal interactions. To address the issue of poor visibility, [9] introduced the FocusAugment model, which leverages blurriness guidance to differentiate between multi-focus and low-focus regions, thereby enhancing the diversity of training samples. Furthermore, late-breaking studies have shifted towards addressing extreme underwater degradation. Recent models such as [10] employ a cross-scale interaction strategy to maintain boundary integrity under severe light attenuation. The method proposed in [11] optimizes multimodal feature interaction and enhancement through a two-stage training strategy. It further introduces a cross-scale learning strategy to promote coarse-to-fine feature fusion, thereby alleviating indistinct object boundaries in underwater vision tasks. By incorporating these hierarchical and attention-based strategies, contemporary methods have significantly reduced the uncertainty inherent in underwater multi-sensor data. However, optical distortions, water patterns, and the lack of geometric information limit the practical applications of RGB-based methods [12,13]. Incorporating depth information into the underwater object detection process has proven to be an effective solution [14–16]. Depth images, compared to RGB images, provide valuable information about object shapes, depths, and other geometric features [17–20]. These geometric features are particularly useful in underwater scenarios, as they help address occlusion issues and low visibility. By combining complementary information from depth and RGB images, more comprehensive underwater object detection can be achieved [21–29].

Despite its advantages, fully leveraging the complementary nature of RGB and depth images remains a challenging task due to their inherent differences [30–35]. Additionally, depth images may contain substantial noise due to the limited detection range and imaging conditions of depth cameras. [36–38] Traditional methods primarily focus on effectively fusing multimodal information using dual-branch architectures to extract RGB and depth image features separately [39–42]. While these approaches provide solid solutions for multimodal information fusion, they struggle to ensure the effective alignment of RGB and depth information [43–46], especially in complex underwater environments. Attempts to reduce sensitivity to depth information have been made, but implicit interactions between RGB features and depth information often lead to reduced model efficiency [47–51]. To summarize, contemporary underwater RGB-D fusion frameworks are hindered by three major challenges. First, underwater depth maps often suffer from quality degradation caused by backscattering and sensor limitations, reducing the reliability of cross-modal guidance. Second, significant modality discrepancies between RGB and depth images introduce severe spatial and channel-wise alignment ambiguity, limiting effective information interaction. Third, existing attention mechanisms generally compress either spatial or channel dimensions, leading to cross-dimensional context loss and insufficient modeling of dense global correlations required for accurate boundary perception.

In this paper, we propose an Information Cross-Fusion Network (ICFN) for multimodal fusion in underwater object detection. Our model introduces cross-attention feature injection (CAFI) and information embedding (IEM) modules to achieve significant improvements in RGB and depth information fusion. Specifically, CAFI module precisely calibrates the features of different modalities at spatial and channel levels, fully leveraging the complementary nature of modalities. Moreover, IEM module combines channel attention and spatial attention to efficiently encode global features and support multi-scale learning. This design enhances the network’s robustness against depth noise while significantly improving the accuracy of feature alignment and fusion. The main contributions of this paper are as follows:

1. We present an information cross-fusion network for underwater object detection, which utilizes a dual-branch architecture to capture multi-modal information while effectively mitigating the inherent noise in depth images.
2. The proposed information embedding module employs a dual attention mechanism, interleaving spatial and channel attention, to extract critical features.
3. The cross-attention feature injection module facilitates the effective integration of RGB and depth features through global pooling.

## Proposed method

### Architecture overview

**Fig 1:**
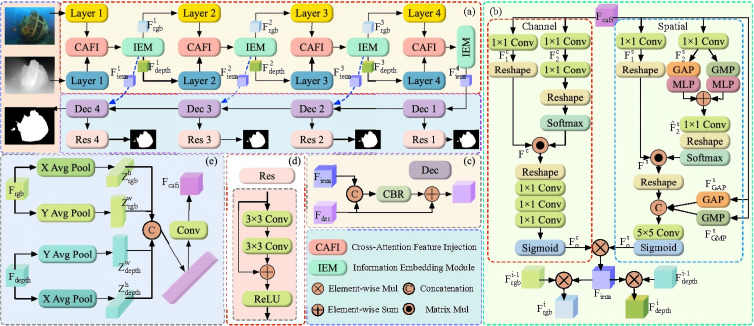
The overall framework of our proposed ICFN model. (a) Overall architecture. (b) IEM. (c) Decoding module. (d) Residual module. (e) CAFI.

We propose a CAFI and an IEM to extract and integrate salient cues from RGB and depth images. These modules are strategically embedded across multiple scales within the architecture to ensure robust feature fusion and refinement. As shown in Fig 1, the proposed framework comprises three core components. The dual-branch encoder independently extracts features from RGB and depth modalities, with ResNet50 serving as the backbone network (Fig 1(a)). The CAFI (Fig 1(e)) facilitates precise feature alignment and interaction across modalities, mitigating misalignment issues while enhancing the complementary fusion of RGB and depth features. Meanwhile, the IEM (Fig 1(b)) captures multi-level attention features, preserving both spatial and channel dependencies, and seamlessly integrates them with the initial feature representations to improve segmentation accuracy.

### Cross-attention feature injection module

While RGB and depth images have the potential to complement each other and boost feature representation in various tasks, the inherent noise in depth maps, caused by light scattering, water turbidity, and sensor limitations, can negatively impact network performance when directly fused with RGB features. To mitigate these challenges, we propose a CAFI to enable effective interaction between multimodal features, as illustrated in Fig 1(e). Unlike conventional global average pooling that collapses the entire spatial distribution into a single descriptor, the proposed directional pooling strategy preserves anisotropic structural responses along horizontal and vertical directions. Since underwater backscattering noise is typically locally distributed and spatially inconsistent, directional average pooling statistically suppresses these high-frequency perturbations while maintaining long-range structural continuity of salient objects. Furthermore, the CAFI does not rely solely on pooled depth features. Instead, RGB features with clearer structural boundaries are jointly injected through cross-modal fusion and recalibrated using a 1×1 convolution. This process enables the network to recover object boundary consistency and compensate for the potential over-smoothing effect introduced by average pooling, thereby achieving more robust multimodal feature interaction under underwater degradation.

This module performs one-dimensional feature encoding to aggregate information along both horizontal and vertical dimensions. By doing so, it captures long-range dependencies across spatial dimensions, facilitating precise localization of key objects. Specifically, given the RGB feature map $F_{rgb}\in {\mathbb{R}}^{C\times H\times W}$ and the depth feature map $F_{depth}\in {\mathbb{R}}^{C\times H\times W}$, we utilize an average pooling operation to encode and align features along horizontal and vertical axes, respectively:


$$\begin{array}{c} \begin{array}{cc} & Z_{rgb}^{h}={𝒳}_{avgpool}(F_{rgb}),Z_{rgb}^{w}={𝒴}_{avgpool}(F_{rgb})\\ & Z_{depth}^{h}={𝒳}_{avgpool}(F_{depth}),Z_{depth}^{w}={𝒴}_{avgpool}(F_{depth}) \end{array}, \end{array}$$ (1)

where, ${𝒳}_{avgpool}$ and ${𝒴}_{avgpool}$ refer to the average pooling operations applied along the vertical and horizontal dimensions, respectively. To clarify, the feature map $Z^{h}$ is generated by aggregating information along the vertical dimension (resulting in *H* = 1), while $Z^{w}$ is obtained by aggregating along the horizontal dimension (*W* = 1). Once these directional features are extracted, we combine all four features and process them through a convolutional layer to produce the final output representation:


$$\begin{array}{c} F_{cafi}={Conv}^{1\times 1}\left(\left[Z_{rgb}^{h},Z_{rgb}^{w},Z_{depth}^{h},Z_{depth}^{w}\right]\right), \end{array}$$ (2)

where, [·,·,·,·] signifies the concatenation operation along the spatial axis, while $F_{cafi}\in {\mathbb{R}}^{C\times 2(H+W)}$ denotes the resulting spatial feature map.

### Information embedding module

Existing attention mechanisms often compress spatial or channel dimensions, potentially leading to the loss of contextual information. Furthermore, isolating the learning of spatial or channel attention without considering their interdependence limits the model’s capacity for feature representation. To overcome these challenges, we introduce an IEM that integrates spatial and channel attention directly into multimodal feature maps, producing attention features that match the original feature map dimensions. As illustrated in Fig 1(b), this module incorporates two complementary mechanisms: spatially embedded channel attention and channel-embedded spatial attention. By cross-learning these two attention types, the module effectively combines spatial and channel encodings to harness their strengths for improved performance. Moreover, the proposed module enables simultaneous computation of spatial and channel attention without collapsing one dimension to accommodate the other.

In traditional channel attention mechanisms, spatial information is often compressed using global average pooling or global max pooling. However, this compression approach limits the ability to represent spatial dimensions comprehensively, resulting in suboptimal feature modeling. To address this limitation, we propose integrating spatial attention into channel attention for enhanced global context modeling. As shown in Fig 1(b), the input multimodal feature $F_{cafi}$ is first processed through two 1×1 convolutional layers, producing two feature maps, $F_{1}^{c}$ and $F_{2}^{c}$, both with dimensions ${\mathbb{R}}^{C\times H\times W}$. The dimensions of $F_{1}^{c}$ are reshaped to ${\mathbb{R}}^{C\times HW}$, while $F_{2}^{c}$ undergoes transformation into a single-channel feature map via a 1×1 convolution. Subsequently, the features are normalized using the Softmax function, and a matrix multiplication is performed between $F_{1}^{c}$ and $F_{2}^{c}$ to derive the spatial embedding matrix, formally expressed as:


$$\begin{array}{c} F^{c}=F_{1}^{c}⊗F_{2}^{c},F^{c}\in {\mathbb{R}}^{C\times 1},F_{1}^{c}\in {\mathbb{R}}^{C\times HW},F_{2}^{c}\in {\mathbb{R}}^{HW\times 1}. \end{array}$$ (3)

The resulting feature map $F^{c}$ is then processed through three sequential 1×1 convolutional layers to refine its channel-wise representation. To summarize, the operations in the channel attention branch can be formally represented as:


$$\begin{array}{c} F_{o}^{c}=\sigma \left({Conv}_{\times 3}^{1\times 1}\left(F^{c})\right)\right), \end{array}$$ (4)

where, σ(·) represents the Sigmoid activation function. From Equation (3), it can be observed that the channel attention branch output integrates $F_{1}^{c}$ and $F_{2}^{c}$. Unlike traditional methods that directly compress spatial information, we abstract spatial information into a feature matrix, enabling it to be seamlessly incorporated into the channel attention map through a learnable process.

In the spatial attention branch, the input feature $F_{cafi}$ is first fed into two 1×1 convolutional layers, producing two feature maps: $F_{1}^{s}\in {\mathbb{R}}^{C\times H\times W}$ and $F_{2}^{s}\in {\mathbb{R}}^{C\times H\times W}$. The feature map $F_{1}^{s}$ is then reshaped to ${\mathbb{R}}^{C\times HW}$, while $F_{2}^{s}$ undergoes global average pooling and global max pooling to extract spatial information. The resulting pooled features are refined using an MLP and subsequently combined via element-wise addition. Finally, a 1×1 convolution is applied to fuse the features derived from the different pooling strategies. The computation can be formally expressed as:


$$\begin{array}{c} {\hat{F}}_{2}^{s}={Conv}^{1\times 1}\left(MLP\left(GAP\left(F_{2}^{s}\right)\right)+MLP\left(GMP\left(F_{2}^{s}\right)\right)\right), \end{array}$$ (5)

where ${\hat{F}}_{2}^{s}\in {\mathbb{R}}^{C\times 1\times 1}$ denotes the channel-wise feature descriptor. To normalize ${\hat{F}}_{2}^{s}$ to the range [0,1], we employ the Softmax function.

The channel information embedding matrix $F^{s}$ is then obtained via matrix multiplication:


$$\begin{array}{c} F^{s}=F_{1}^{s}⊗{\hat{F}}_{2}^{s}, \end{array}$$ (6)

where $F_{1}^{s}\in {\mathbb{R}}^{HW\times C}$ is reshaped from the original feature map. Prior to matrix multiplication, ${\hat{F}}_{2}^{s}$ is implicitly flattened along the channel dimension. The resulting feature $F^{s}\in {\mathbb{R}}^{HW\times 1}$ encodes the aggregated channel information.

The reshaped feature map $F^{s}$ has dimensions ${\mathbb{R}}^{1\times H\times W}$. To enhance spatial attention, we further include features derived from global average pooling (GAP) and global max pooling (GMP), denoted as $F_{GAP}^{s}\in {\mathbb{R}}^{1\times H\times W}$ and $F_{GMP}^{s}\in {\mathbb{R}}^{1\times H\times W}$, respectively, with their channel dimensions reduced to a single channel. These features are then fused using a 5×5 convolutional layer, and the final spatial attention map is obtained by applying the Sigmoid activation function. The overall computation is formally represented as:


$$\begin{array}{c} F_{o}^{s}=\sigma \left({Conv}^{5\times 5}Concat\left(F^{s},F_{GAP}^{s},F_{GMP}^{s}\right)\right). \end{array}$$ (7)

To compress and learn features across multiple dimensions, we utilize convolutional layers. However, as global pooling inherently discards significant spatial information, we integrate $F_{GAP}^{s}$ and $F_{GMP}^{s}$ to restore and complement the lost spatial context. As outlined in Equations (6) and (7), we extract global channel-level contextual information and inject channel attention features into the spatial attention map at each location, leveraging dense global correlations to merge the feature vectors across channels.

By uniting the outputs of the two branches, the module provides a richer and more holistic set of attention features, which significantly improves segmentation accuracy.


$$\begin{array}{c} F_{iem}=F_{o}^{s}⊗F_{o}^{c}. \end{array}$$ (8)

The feature map $F_{iem}$ is forwarded to the decoder via skip connections. Simultaneously, $F_{iem}$ is fused with $F_{rgb}$ and $F_{depth}$ and then propagated back to the respective encoders, emphasizing critical features for enhanced representation.


$$\begin{array}{c} F_{rgb}^{i}=F_{rgb}^{i-1}⊗F_{iem},F_{depth}^{i}=F_{depth}^{i-1}⊗F_{iem}. \end{array}$$ (9)

### Loss function

To enhance multi-scale feature learning and facilitate stable optimization, we employ a deep supervision strategy by generating four saliency maps $\{Res_{i}{\}}_{i=1}^{4}$ from different decoder stages, as illustrated in Fig 1(a). The overall training objective is defined as:


$$\begin{array}{c} L_{total}=\sum_{i=1}^{4}\lambda_{i}\cdot L_{stage}^{i}, \end{array}$$ (10)

where $\lambda_{i}$ denotes the weight for each stage. Following common practice, we set $\lambda_{i}=1$ for all stages. $L_{stage}^{i}$ represents the discrepancy between the *i*-th prediction and the ground truth. For each stage, we combine the Binary Cross-Entropy (BCE) loss and the Intersection over Union (IoU) loss:


$$\begin{array}{c} L_{stage}^{i}=L_{bce}(P^{i},G)+L_{iou}(P^{i},G), \end{array}$$ (11)

where $P^{i}\in [0,1{]}^{H\times W}$ denotes the sigmoid-normalized saliency prediction from the *i*-th stage and $G\in \{0,1{\}}^{H\times W}$ is the corresponding ground truth. The pixel-wise BCE loss is defined as:


$$\begin{array}{c} L_{bce}(P^{i},G)=-\frac{1}{H\times W}\sum_{x,y}\left[G_{x,y}\log(P_{x,y}^{i}+\epsilon )+(1-G_{x,y})\log(1-P_{x,y}^{i}+\epsilon )\right], \end{array}$$ (12)

where $\epsilon =10^{-7}$ is a small constant to avoid numerical instability. To further enhance structural consistency, we adopt the IoU loss to measure the global overlap between prediction and ground truth:


$$\begin{array}{c} L_{iou}(P^{i},G)=1-\frac{\sum_{x,y}(P_{x,y}^{i}\cdot G_{x,y})+\epsilon }{\sum_{x,y}(P_{x,y}^{i}+G_{x,y}-P_{x,y}^{i}\cdot G_{x,y})+\epsilon }. \end{array}$$ (13)

By integrating these two complementary terms, the proposed loss function effectively balances pixel-wise accuracy and global structural consistency, enabling the network to capture both fine-grained details and coherent object-level structures.

## Experiments

### Dataset

The USOD10K dataset is a large-scale benchmark tailored for underwater salient object detection, consisting of 10,255 underwater images spanning 12 distinct underwater scenarios and 20 salient object categories. These scenarios cover diverse underwater conditions, including varying levels of turbidity, illumination, and object occlusion, reflecting real-world complexities. The inclusion of 20 object categories, ranging from marine organisms to man-made structures, ensures a broad coverage of salient object types typically encountered underwater. Each image is paired with its corresponding depth map and boundary annotations, offering a multimodal perspective that supports robust feature learning and performance evaluation.

### Implementation details

Our experiments were conducted on the USOD10K dataset. During training, RGB and depth image pairs were randomly cropped into patches of size 140×140. Parameter updates were performed using the Adam optimizer with a batch size of 4 and an initial learning rate of $1\times 10^{-4}$. The learning rate was progressively reduced using a cosine decay strategy, reaching a minimum of $1\times 10^{-6}$ by the end of training. For a fair comparison, all deep learning-based methods were retrained on the USOD10K dataset under their default configurations, and results were evaluated using a unified evaluation framework.

### Evaluation metrics

To comprehensively evaluate model performance, we employ the following four key metrics. Structural Similarity Measure ($S_{\alpha }$) measures the structural resemblance between the predicted segmentation map and the ground truth by combining luminance, contrast, and structural components.


$$\begin{array}{c} S_{\alpha }=\alpha S_{o}+(1-\alpha )S_{r}, \end{array}$$ (14)

where, $S_{o}$ and $S_{r}$ represent the object-aware and region-aware structural similarity, respectively, with α set to 0.5.

The weighted F-measure ($F_{\beta }^{\omega }$) balances precision and recall by introducing a weighting factor, effectively mitigating the impact of class imbalance within the dataset.


$$\begin{array}{c} F_{\beta }^{\omega }=(1+\beta^{2})\frac{P^{\omega }R^{\omega }}{\beta^{2}P^{\omega }+R^{\omega }}, \end{array}$$ (15)

where, β represents the effectiveness of the detection, $P^{\omega }$ denotes the weighted precision, and $R^{\omega }$ denotes the weighted recall.

Adaptive E-measure ($E_{\xi }$) captures both fine-grained pixel-level details and high-level image-level consistency.


$$\begin{array}{c} E_{\xi }=\frac{1}{w\times h}\sum_{x=1}^{w}\sum_{y=1}^{h}\Phi (x,y), \end{array}$$ (16)

where, *h* and *w* represent the height and width of the segmented result image, respectively. Φ is the enhanced alignment matrix.

Mean Absolute Error (*MAE*) offering an intuitive reflection of prediction deviations and the model’s overall error magnitude.


$$\begin{array}{c} MAE=\frac{1}{n}\sum_{i=1}^{n}|y_{i}-{\hat{y}}_{i}|, \end{array}$$ (17)

where, $y_{i}$ represents the ground truth, and ${\hat{y}}_{i}$ represents the predicted value.

### Experimental results

#### Comparison with SOTA method.

We conducted a comprehensive comparison of our proposed method against 16 state-of-the-art methods. Both quantitative evaluation and qualitative analysis consistently highlight the superior performance of our approach. (F3Net [52], DANet [53], DASNet [54], BTSNet [21], MFNet [39], HAINet [55], D3Net [56], PSGLoss [57], CSNet [12], TCUSOD [58], CrossFuse [1], LESOD [59], HENet [60], MAGNet [61], CATNet [62], CPNet [63]).

As shown in Table 1, our method achieves the best performance on the $S_{\alpha }$ and the $E_{\xi }$, underscoring its ability to capture both global image structure and fine-grained details. This is primarily due to the introduction of a cross-fusion attention mechanism, which dynamically emphasizes salient object regions while suppressing background noise. Such a mechanism proves particularly effective in complex underwater conditions, addressing issues like uneven illumination, occlusions, and boundary ambiguity. For the $F_{\beta }^{\omega }$, our method outperforms approaches like CSNet, highlighting its strength in achieving a precise balance between precision and recall. Additionally, the integration of depth information significantly enhances the model’s robustness in detecting underwater salient objects, contributing to its superior Mean Absolute Error (*MAE*) performance on the USOD10K dataset.

**Table 1:** Performance comparison on USOD10k. The best indicator is shown in bold.

Methods	$S_{\alpha }$ ↑	$E_{\xi }$ ↑	$F_{\beta }^{\omega }$ ↑	*MAE* ↓
**F3Net**	0.9140	0.9599	0.9171	0.0251
**DANet**	0.9006	0.9449	0.8934	0.0279
**DASNet**	0.9199	0.9504	0.9202	0.0247
**BTSNet**	0.9093	0.9542	0.9104	0.0291
**MFNet**	0.8425	0.9146	0.8193	0.0512
**HAINet**	0.9101	0.9445	0.9102	0.0233
**D3Net**	0.9001	0.9422	0.8881	0.0302
**PSGLoss**	0.8640	0.9078	0.8508	0.0417
**CSNet**	0.8990	0.9102	0.8870	0.0490
**TCUSOD**	0.9215	0.9683	0.9236	0.0201
**CrossFuse**	0.9121	0.9585	0.9176	0.0288
**LESOD**	0.9131	0.9307	0.8797	0.0282
**HENet**	0.9149	0.9330	0.8842	0.0275
**MAGNet**	0.9168	0.9358	0.8859	0.0268
**CATNet**	0.9319	0.9164	0.8633	0.0304
**CPNet**	**0.9225**	0.8762	0.7766	0.0423
**Our**	0.9240	**0.9685**	**0.9313**	**0.0182**

**Fig 2:**
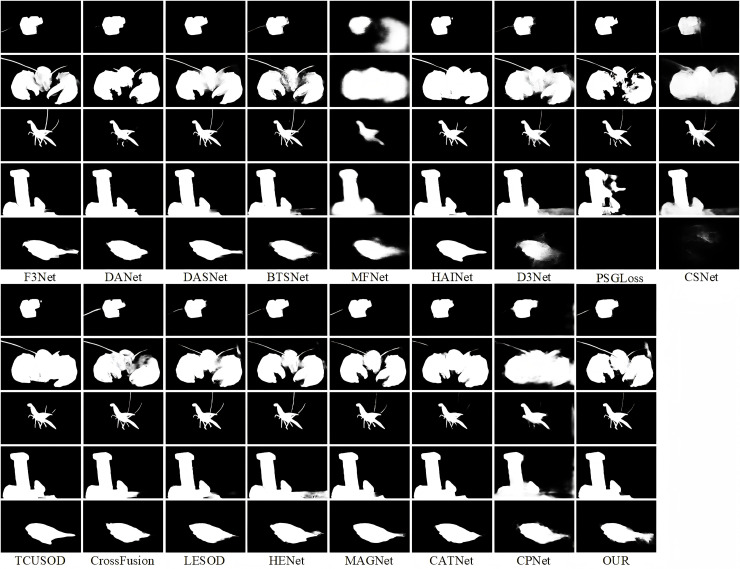
Qualitative analysis results.

Fig 2 illustrates the qualitative performance of our proposed method compared with various cutting-edge approaches on the USOD10K dataset. The results clearly demonstrate that our method consistently outperforms others across diverse underwater scenes and salient object categories. Our method’s segmentation outputs exhibit a high degree of agreement with the ground truth (GT), particularly excelling in target region completeness and boundary precision. For instance, our method effectively preserves the integrity of object regions while minimizing omissions and overflows (as seen in the first and second rows of Fig 2). In more challenging underwater conditions, the proposed method produces smoother and more precise object boundaries, significantly improving the retention of fine-grained details (Fig 2, third and fourth rows). In contrast, methods like PSGLoss and CrossFusion struggle to suppress background noise, resulting in confusion between target objects and the background. By leveraging advanced feature extraction and deep multimodal information fusion, our method achieves a clear separation between salient objects and the background, yielding more accurate and reliable segmentation results.

#### Complexity comparison.

To evaluate the computational efficiency of the proposed method, we compare the number of parameters, FLOPs, and inference speed (FPS) with several representative RGB-D SOD models, as reported in Table 2. The proposed model contains 308.58M parameters and requires 20.63G FLOPs. Although our method introduces more parameters than existing lightweight models and several recent RGB-D SOD approaches, it achieves the lowest FLOPs among the compared methods. In particular, the proposed network reduces computational cost compared with CATNet (29.11G FLOPs) and CPNet (25.16G FLOPs), demonstrating that the designed feature interaction strategy can effectively enhance representation capability without introducing excessive computational overhead. In terms of inference speed, our method achieves 35.16 FPS, which is comparable to CATNet (36.47 FPS) and substantially faster than CPNet (21.35 FPS). These results indicate that the proposed network achieves a favorable trade-off between detection accuracy and computational efficiency. Considering its superior detection performance together with competitive FLOPs and inference speed, the proposed method provides an effective solution for RGB-D salient object detection.

**Table 2:** Complexity comparison of various models.

Methods	Params (M) ↓	FLOPs (G) ↓	FPS ↑
**LESOD**	2.8523	0.4073	255.49
**HENet**	10.4258	1.5664	35.58
**MAGNet**	16.0661	1.4052	32.10
**CATNet**	262.7368	29.1124	36.47
**CPNet**	216.5000	25.1582	21.35
**Our**	308.5757	20.6325	35.16

### Ablation study

The performance analysis of our proposed modules is summarized in Table 3. The Base model serves as the foundation, consisting of a ResNet-based dual-branch encoder-decoder architecture. The incremental addition of the decoder module (Dec.), IEM, and CAFI demonstrates the individual contributions of each module to the overall performance. Please note that the structure of the decoder module includes Fig 1(c) and 1(d). The baseline model alone delivers subpar segmentation results, particularly struggling with feature reconstruction and complex object boundaries. The improved decoder module enhances feature recovery but shows limited ability to refine intricate edge details. The introduction of the IEM effectively incorporates richer global and contextual information, significantly improving the representation of salient regions, thereby boosting structural similarity and detection accuracy. When CAFI is added, the network excels in modeling dependencies between RGB and depth features, reinforcing the separation of salient objects from background noise. This module enhances both structural integrity and fine-detail retention, further improving segmentation results. Finally, multi-scale supervision reduces errors across all evaluation metrics, demonstrating the network’s ability to achieve highly precise salient object detection while significantly mitigating the impact of noise in underwater environments.

**Table 3:** Performance comparison of different modules.

Methods	$S_{\alpha }$ ↑	$E_{\xi }$ ↑	$F_{\beta }^{\omega }$ ↑	*MAE* ↓
**Base ICNet**	0.8403	0.9058	0.8585	0.0586
**Base ICNet(+Dec.)**	0.8628	0.9187	0.8731	0.0542
**Base ICNet(+Dec. + IEM)**	0.8996	0.9369	0.9005	0.0251
**Base ICNet(+Dec. + IEM+CAFI)**	0.9145	0.9551	0.9146	0.0210
**ICFN**	0.9240	0.9685	0.9313	0.0182

### Ablation study on IEM

To evaluate the efficacy of the proposed IEM, we conducted a series of ablation experiments by isolating its internal components and comparing them with the established CBAM module. The quantitative results are summarized in Table 4. The results indicate that the standard CBAM, which employs a sequential spatial and channel attention structure, achieves an $S_{\alpha }$ of 0.8712. Our individual components—the Channel Branch ($S_{\alpha }=0.8768$) and the Spatial Branch ($S_{\alpha }=0.8867$)—already exhibit superior performance compared to CBAM, suggesting that our specific design for underwater feature embedding is more robust than traditional attention mechanisms. The integration of both branches into the full IEM leads to a significant performance leap, achieving the highest $S_{\alpha }$ of 0.9240 and $E_{\xi }$ of 0.9685. Compared to the Spatial Branch alone, the full IEM reduces the *MAE* significantly from 0.0301 to 0.0182. This sharp improvement demonstrates that our interlocking mechanism—which mutually embeds spatial and channel information—creates a crucial synergistic effect. By leveraging cross-dimensional correlations, the IEM effectively suppresses persistent backscattering noise and recalibrates object boundaries, producing much sharper and more accurate saliency maps than simple additive or sequential attention structures like CBAM.

**Table 4:** Ablation study on IEM.

Methods	$S_{\alpha }$ ↑	$E_{\xi }$ ↑	$F_{\beta }^{\omega }$ ↑	*MAE* ↓
**w/ CBAM**	0.8712	0.9188	0.8795	0.0498
**w/ Channel Branch**	0.8768	0.9134	0.8909	0.0431
**w/ Spatial Branch**	0.8867	0.9231	0.9001	0.0301
**w/ IEM**	0.9240	0.9685	0.9313	0.0182

#### Ablation study on hierarchical design.

To justify the rationality of the four-tier (Layers 1–4) hierarchical design in ICFN, we conducted ablation experiments by varying the network depth, as summarized in Table 5. The results reveal a clear evolutionary trend in feature representation. The 2-Layer and 3-Layer variants yield sub-optimal performance, particularly in terms of *MAE* and $E_{\xi }$. This is attributed to the limited receptive fields of shallower architectures, which lack the high-level semantic guidance necessary to distinguish salient objects from heavy underwater backscattering noise. Specifically, the four-tier design enables a collaborative mapping between spatial fineness and semantic robustness. The deepest layers (Layers 3–4) capture abstract, global semantic anchors that are essential for robust object localization (reflected in the significant rise of $S_{\alpha }$), while the shallower layers (Layers 1–2) provide the high-resolution spatial details required for boundary refinement. The transition from a 3-layer to a 4-layer structure results in a drastic *MAE* reduction to 0.0182, confirming that Layer 4 is the “sweet spot” for bridging the gap between low-level textures and high-level global context. This hierarchical evolution ensures that the ICFN achieves a synergistic balance, maintaining both structural integrity and precise edge delineation in complex underwater environments.

**Table 5:** Ablation study on hierarchical network depth.

Methods	$S_{\alpha }$ ↑	$E_{\xi }$ ↑	$F_{\beta }^{\omega }$ ↑	*MAE* ↓
**2-Layer (L1-L2)**	0.8511	0.9099	0.8890	0.05611
**3-Layer (L1-L3)**	0.8901	0.9434	0.9099	0.0311
**4-Layer (ICFN)**	0.9240	0.9685	0.9313	0.0182

#### Limitation analysis.

**Fig 3:**
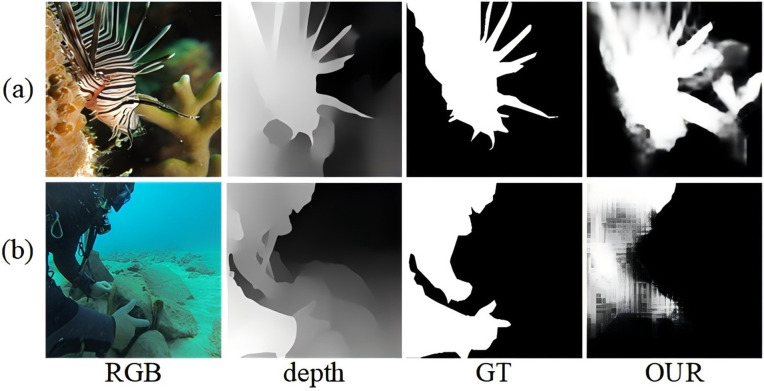
Two representative failure cases of our model.

While the proposed ICFN achieves competitive performance across standard benchmarks, it still exhibits certain limitations in extremely degraded underwater environments, as illustrated by two representative failure cases in Fig 3.

Case 1 (Fig 3(a)) – Saliency Boundary Overflow. In this scenario, the target is heavily camouflaged within a low-contrast and cluttered background in the RGB image, while the foreground boundary and surrounding obstacles are closely attached and exhibit highly similar geometric characteristics in the depth map. Although the proposed framework is designed to exploit long-range spatial dependencies and complementary multimodal information, the severe ambiguity between foreground and background makes reliable discrimination particularly challenging. Consequently, misleading contextual responses may be introduced during feature aggregation and multimodal fusion, causing the predicted saliency region to slightly extend beyond the true object boundary. This failure case indicates that the proposed model still encounters difficulties in accurately separating complex foreground-background structures when spatial and geometric boundaries are heavily entangled.

Case 2 (Fig 3(b)) – Partial Attenuation and Context Blending. This example represents a more challenging situation in which both RGB and depth modalities suffer from severe degradation. As observed in the depth map, the salient diver exhibits substantial structural omission and is degraded into an ambiguous gradient distribution, while the surrounding reef regions generate strong but misleading geometric responses. Meanwhile, underwater scattering significantly weakens the semantic contrast of the target in the RGB image. Consequently, the degraded depth information may provide inaccurate structural guidance during multimodal fusion, while the weakened visual cues further increase ambiguity in target localization. The combined effect of these unreliable cross-modal cues results in an incomplete saliency prediction, where only part of the target is activated and false-positive responses remain in the surrounding background regions. This failure case suggests that the network remains vulnerable when different modalities are degraded in highly asymmetric ways and still lacks sufficient capability to adaptively assess the reliability of conflicting cross-modal information under extreme underwater conditions.

These failure cases suggest that current underwater RGB-D salient object detection models remain partially constrained by adverse imaging conditions and the risk of error propagation across modalities. To address these limitations, future work will focus on incorporating dynamic data-quality evaluation strategies to adaptively down-weight unreliable modalities, introducing modality-reliability assessment mechanisms to improve robustness under sensor degradation, and designing stronger boundary-preserving constraints to further suppress background leakage. We believe these directions are promising for enhancing the robustness and generalization ability of underwater multimodal saliency detection systems.

## Conclusion

In this paper, we introduced the ICFN with two novel components: the CAFI and the IEM. These components effectively harness the complementary nature of RGB and depth information, achieving precise underwater salient object detection through enhanced feature interaction and representation. By incorporating cross-scale learning strategies and deep supervision, our approach captures salient features across multiple scales, significantly improving detection accuracy and robustness. Experimental results on the USOD dataset demonstrate substantial performance gains, showcasing the effectiveness and superiority of the proposed method in addressing underwater salient object detection challenges. Despite its superior performance, the model has certain limitations. The dual-branch interaction and multi-scale attention mechanisms lead to relatively high computational complexity, posing challenges for real-time deployment on hardware-constrained underwater platforms. Furthermore, the model’s efficacy remains somewhat sensitive to the initial quality of the depth maps in extremely turbid environments. In the future, we aim to explore lightweight architecture designs and model compression techniques to improve inference efficiency without sacrificing accuracy.
